# Heat Generation and Transfer Behaviors of Ti-Coated Carbon Steel Rod Adaptable for Ablation Therapy of Oral Cancer

**DOI:** 10.3390/jfb4010027

**Published:** 2013-02-18

**Authors:** Takashi Naohara, Hiromichi Aono, Tsunehiro Maehara, Hideyuki Hirazawa, Shinya Matsutomo, Yuji Watanabe

**Affiliations:** 1 Graduate School of Science Engineering Ehime University Matsuyama 790-8577 Japan; E-Mails: aono.hiromichi.mf@ehime-u.ac.jp (H.A.); maehara.tsunehiro.mg@ehime-u.ac.jp (T.M.); 2 Department of Environmental Materials Engineering, Niihama National College of Technology, Niihama 792-8580, Japan; E-Mail: hirazawa@mat.niihama-nct.ac.jp; 3 Department of Electronic Control Engineering, Niihama National College of Technology, Niihama 792-8580, Japan; E-Mail: shin@ect.niihama-nct.ac.jp; 4 Department of Surgery, Graduate School of Medicine, Ehime University, Toon 791-0295, Japan; E-Mail: yuji@m.ehime-u.ac.jp

**Keywords:** cancer therapy, ablation treatment, high-frequency induction technique, tissue-mimicking phantom, heat transfer simulation, magnetic flux direction

## Abstract

For the purpose of developing a novel ablation therapy for oral cancer, the heat generation and transfer properties of a Ti-coated carbon steel rod with 20-mm length and 1.8-mm outer diameter were investigated by means of a high-frequency induction technique at 300 kHz. The heat generation measurement performed using water (15 mL) revealed that the difference of the inclination angles (θ = 0°, 45° and 90°) relative to the magnetic flux direction only slightly affects the heating behavior, exhibiting the overlapped temperature curves during an induction time of 1200 s. These results suggest that the effect of the shape magnetic anisotropy is almost eliminated, being convenient for the precise control of the ablation temperature in clinical use. In the experiments utilizing a tissue-mimicking phantom, the heat transfer concentrically occurred in the lateral direction for both the planar surface and a 10-mm deep cross-section. However, the former exhibited a considerably lower increase in temperature (ΔT), probably due to the effect of heat dissipation to the ambient air. No significant heat transfer was found to occur to the lower side of the inserted Ti-coated carbon steel rod, which is situated in the longitudinal direction.

## 1. Introduction

Oral cancer is a malignant growth that affects any part of the oral cavity, including the lips, tongue, gums, floor of the mouth and palate [1,2,3]. For patients with oral cancer, the treatment option depends on various factors, such as where the cancer is, its stage and the patient’s general health. As well accepted, there are three main options for the oral cancer treatment [4,5]. Surgical resection is a medical procedure aimed to completely remove the tumor tissue together with adjacent healthy tissue in order to prevent a future cancer relapse [5,6]. However, this option is frequently associated with disfigurement and dysfunction, such as speech impairment, dysphagia and aspiration [7,8]. If these side effects cannot be corrected or minimized, the patients may undergo a substantial decrease in the quality of life. Radiotherapy uses high-energy rays or particles to destroy the cancerous cells. The purpose of this treatment is to destroy cancerous tissues, while preserving the healthy tissue, and the side effects mainly depend on the radiation dosage and the targeted area. The most common side effects displayed by oral cancer patients that undergo radiotherapy are mucositis, taste loss, xerostomia, hyposalivation, *etc*. [9,10,11]. Chemotherapy is usually not used alone to stop oral cancer, but as an adjunct to surgery and/or radiotherapy [12]. In particular, the patients with locally recurrent or metastatic oral cancer receive this treatment, which results in a better survival benefit [13]. This treatment option also has the ability to interfere with the cancerous cell replication; however, their side effects include mucositis oral infections, dysgeusia, and oral bleeding, together with temporary alopecia [14,15,16]. 

In addition to these conventional treatments of oral cancer, the clinical application of a novel option has been desired from the viewpoint of organ conservation in combination with fewer side effects. For patients with oral cancer, it is particularly essential to minimize the cosmetic and functional impairments for a better prognostic quality of life. 

In the present study, we attempted to develop an ablation needle adaptable to the oral cancer therapy utilizing a high-frequency induction technique [17]. It should be emphasized that this thermotherapy is suitable for superficial cancer, due to the easy insertion of the ablation needle into the tumors. Hence, the heat generation and transfer behaviors of a Ti-coated carbon steel rod were experimentally investigated by employing water (15 mL) and a tissue-mimicking phantom. The computer simulation of the heat transfer was also carried out to visualize the temperature distribution in the tissue-mimicking phantom subjected to the insertion of the Ti-coated carbon steel rod.

## 2. Materials and Methods

### 2.1. Preparation of the Sample and Tissue-Mimicking Phantom

Table 1 shows the ingredients used to prepare the tissue-mimicking phantom together with their weights and ratios. As seen in the table, the tissue-mimicking phantom was made of agar, polyethylene powder, sodium chloride (NaCl), boric acid and TX-151 in addition to deionized water [18,19]. Agar was used to hold the phantom shape by preventing separation of the water-soluble contents. The TX-151 was used as a thickener to mix the agar solution and the polyethylene powder, while the boric acid was employed as a preservative. The measured quantities of these ingredients were added to hot deionized water and stirred to make a uniform solution. The mixture was then poured into a plastic container and allowed to cool at room temperature. A small cylindrical section was removed from this square-shaped tissue-mimicking phantom for the following heat transfer experiments.

In a previous paper [20], we reported the heating properties of a carbon steel rod embedded into Ti-tubes of various inner diameters in a high-frequency induction field at 300 kHz. Based on the previous experiments, there was an optimum thickness to minimize the effect of the inclination angle relative to the magnetic flux direction. The optimum Ti-tube had a 1.8-mm outer diameter and 1.0-mm inner diameter, which was used in the present study. A ferromagnetic carbon steel rod with a 20-mm length was embedded in the Ti-tube to form the rod-like specimen. 

**Table 1:** The ingredients, their weights and ratios used for the preparation of the tissue-mimicking phantom in the present study.

Ingredient	Weight (g)	Ratio (%)
Deionized water	365.0	74.8
Agar	20.0	4.1
Polyethylene powder	70.0	14.4
Sodium chloride (NaCl)	7.9	1.6
Boric acid	7.9	1.6
TX-151	16.9	3.5

### 2.2. Experimental Procedures

**Figure 1:**
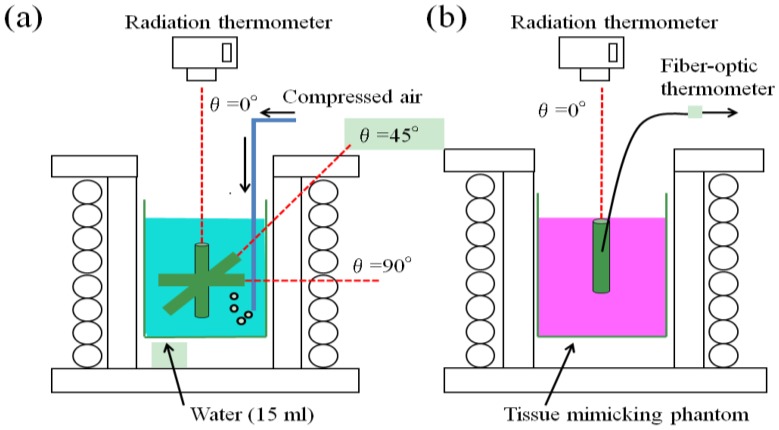
Partial experimental set up showing the specimen placed in a high-frequency induction coil to measure the heating behaviors (**a**) in water (15 mL) and (**b**) tissue-mimicking phantom at different inclination angles relative to the magnetic flux direction.

Figure 1 shows the partial experimental set up around the specimen placed in a high-frequency induction coil for the different measuring conditions. The increase in temperature (ΔT) was measured in water (15 mL) to estimate the heat generation ability using a radiation thermometer at three different angles of θ = 0°, 45° and 90° relative to the magnetic flux direction (Figure 1a). To clarify the heat transfer behavior, the ΔT value of the tissue-mimicking phantom subjected to the θ = 0° specimen insertion was also measured using a radiation thermometer and a fiber-optic thermometer (Figure 1b). The magnetic field and frequency were 1.69 kA/m and 300 kHz, respectively, for both measuring conditions.

**Figure 2:**
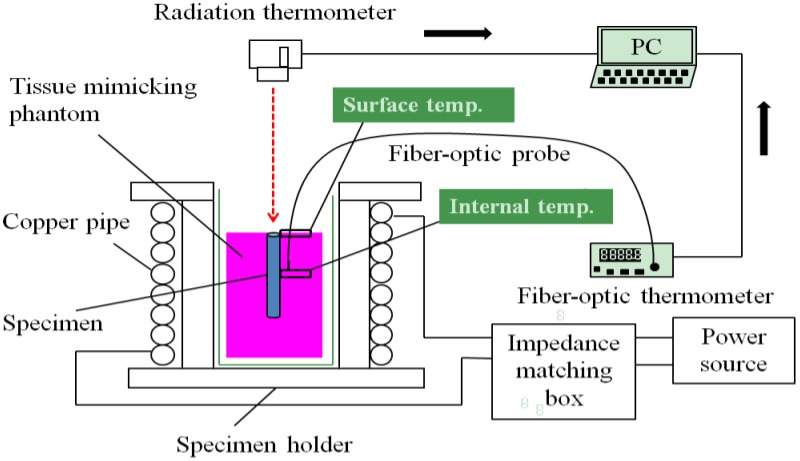
Complete experimental setup for measuring the heat transfer behavior of the tissue-mimicking phantom subjected to the insertion of the Ti-coated carbon steel rod in the high-frequency induction field of 1.69 kA/m at 300 kHz.

Figure 2 shows the entire experimental setup used for the measurement of the heat generation and transfer behaviors in the high-frequency induction field at 300 kHz. The high-frequency induction coil was connected to a power supply through an impedance matching box. The tissue-mimicking phantom subjected to the θ = 0° specimen insertion is schematically illustrated in the figure for a more detailed explanation. The heat transfer behavior was investigated for both the planar surface and the 10-mm deep cross-section of the tissue-mimicking phantom using a radiation thermometer and a fiber-optic thermometer. 

**Figure 3:**
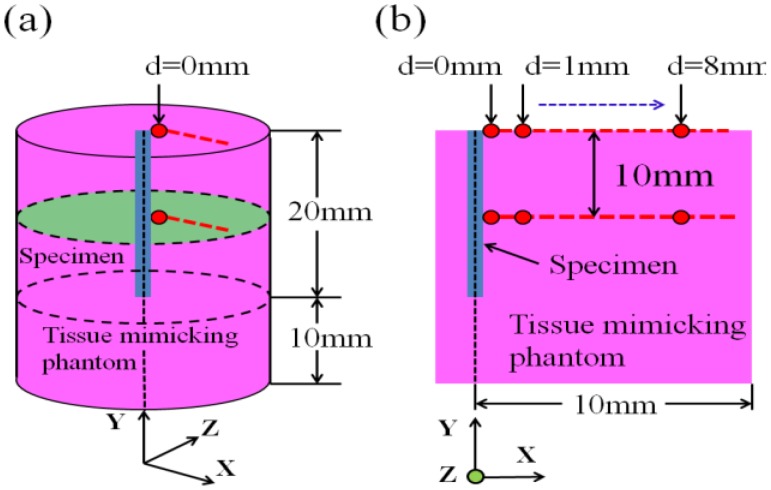
(**a**) Three-dimensional; (**b**) and two-dimensional schematic views of the cylindrical tissue-mimicking phantom subjected to the insertion of the Ti-coated carbon steel rod. The temperature measuring points are denoted by the red circles.

Figure 3 shows the three-dimensional and two-dimensional appearances of the cylindrical tissue-mimicking phantom subjected to the θ = 0° specimen insertion. The specimen with a 1.8-mm outer diameter and 20-mm length was vertically inserted at the center of the planar surface into the cylindrical tissue-mimicking phantom with a 20-mm diameter and 30-mm height. As seen in the figure, the ΔT value was measured from the contact position (d = 0 mm) to a 8-mm distant position (d = 8 mm) at 1-mm intervals for both the planar surface and 10-mm deep cross-section. A fiber-optic thermometer was used to directly measure the inside ΔT value of the tissue-mimicking phantom, as shown in Figure 2.

### 2.3 Computer Simulation of Heat Transfer in the Tissue-Mimicking Phantom

**Figure 4:**
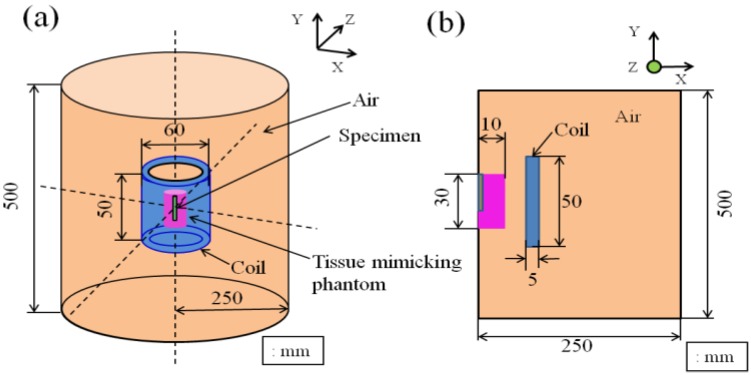
Computer simulation model of heat transfer drawn (**a**) three-dimensionally; (**b**) and two-dimensionally, used in the present study.

The heat transfer behavior was simulated for the tissue-mimicking phantom subjected to the insertion of the Ti-coated carbon steel rod. Heat analysis software, JMAG Studio, ver. 10.0 (JRI Solutions, Ltd., Tokyo, Japan), was employed to visualize the temperature distribution in both the lateral and longitudinal directions. The simulation model was created by considering the physical parameters of the carbon steel rod, Ti-tube and tissue-mimicking phantom, as well as their size, frequency, current condition and turn number of the coil. The relative magnetic permeability value of the ferromagnetic carbon steel rod used in the simulation was 2,000, while this value was 1 for the non-magnetic Ti-tube. The three-dimensional and two-dimensional models used in the present study are presented in Figure 4.

## 3. Results and Discussion

### 3.1. Heat Generation Ability of the Ti-Coated Carbon Steel Rod

**Figure 5:**
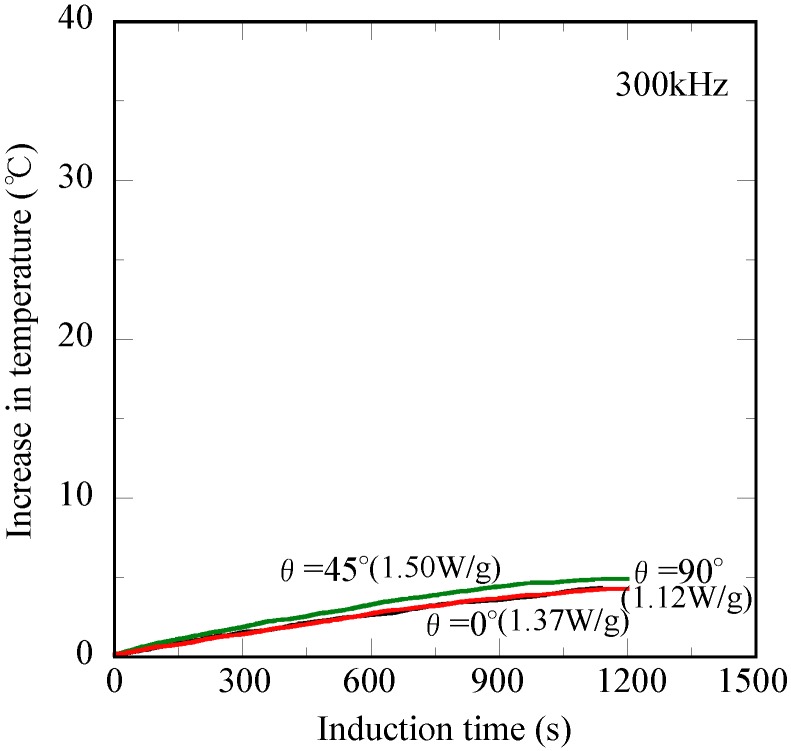
Changes in temperature of water (15 mL) for the Ti-coated carbon steel rod at different inclination angles relative to the magnetic flux direction *versus* the induction time in the high-frequency induction field of 1.69 kA/m at 300 kHz.

Figure 5 shows the relationship between the increase in temperature (ΔT) of water (15 mL) and induction time for the Ti-coated carbon steel rod in the high-frequency field of 1.69 kA/m at 300 kHz. Both the θ = 0° and θ = 90° specimens exhibit overlapping temperature curves that reach the ΔT value of 4.8 °C after 1,200 s. The ΔT similarly increases with the increasing induction time, and its value reached 5.0 °C after 1,200 s for the θ = 45° specimen. It is important to note that the non-oriented heating property relative to the magnetic flux direction is achieved for the measurement in water (15 mL) [20]. In the novel thermotherapy, the injection direction of the ablation needle seems to significantly vary due to the tumor location. A non-oriented heating property relative to the magnetic flux direction is indispensable for the precise control of the treatment temperature. However, the shape magnetic anisotropy, which originates from the demagnetizing field coefficient, results in an undesirable effect on the heating properties in the high-frequency induction field [21]. Thus, the present Ti-coated carbon steel rod appears to have a high potential as the ablation needle for a novel oral cancer treatment [17]. The heat generation ability was calculated using the temperature enhancement ratio during the initial 120 s of the ΔT measurement. These values were 1.50, 1.37 and 1.12 W/g for the θ = 45°, θ = 90° and θ = 0° specimens, respectively, as denoted in the figure. It is noted that the difference in these values only slightly affects the heating behavior of water (15 mL). According to the report by Yamada *et al*. [22], the heating of the cancer cells up to 50 °C for 600 s is sufficient for complete cell killing, and the heat dose of 1.70 W/g is required for a 10-mm size tumor. Considering the influence of the body temperature (approximately 37.0 °C), the ΔT value of 10.0 °C after 1,200 s appears to be a criterion for the effective ablation treatment of oral cancer. It appears that the Ti-coated carbon steel rod used in the present study possesses the potential to be the ablation needle for this novel oral cancer therapy.

### 3.2. Heat Transfer Behavior of the Tissue-Mimicking Phantom

**Figure 6:**
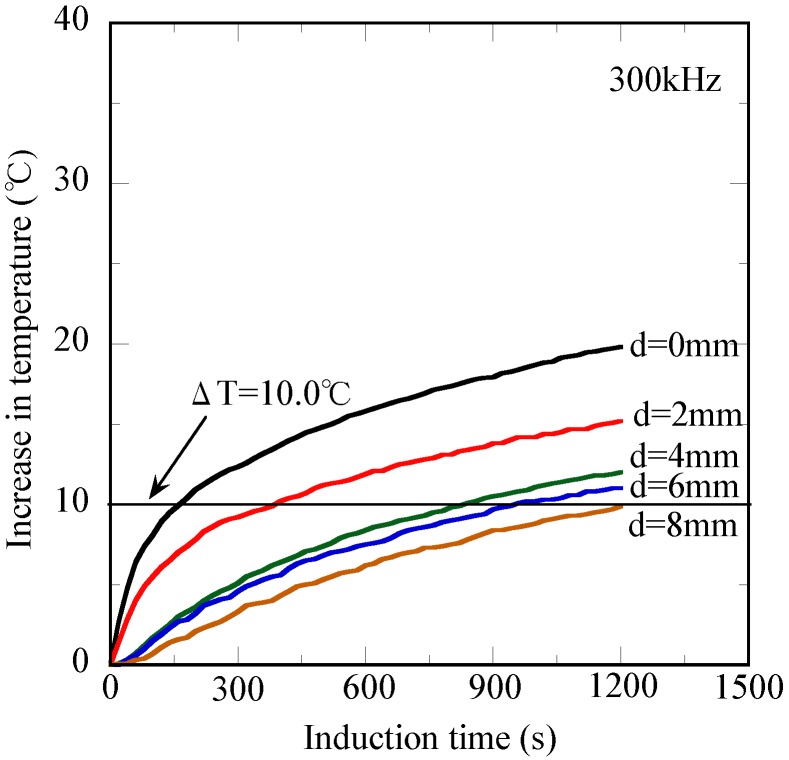
Changes in temperature of the tissue-mimicking phantom with different distances from the contact position *vs.* the induction time at the 10-mm deep cross-section.

Figure 6 shows the induction time dependence of the heat transfer behavior for the tissue-mimicking phantom at the 10-mm deep cross-section. The measured temperature increases continuously with the induction time and reached ΔT = 19.8 °C after 1,200 s at the contact position (d = 0 mm). A similar induction time dependence of ΔT is obtained from the other temperature curves; however, the ΔT after 1,200 s gradually decreases with the increasing distance from the contact position (d = 0 mm). For instance, the ΔT values after 1,200 s are 12.0 ^o^C and 9.9 ^o^C at the d = 4 mm position and d = 8 mm position, respectively.

**Figure 7:**
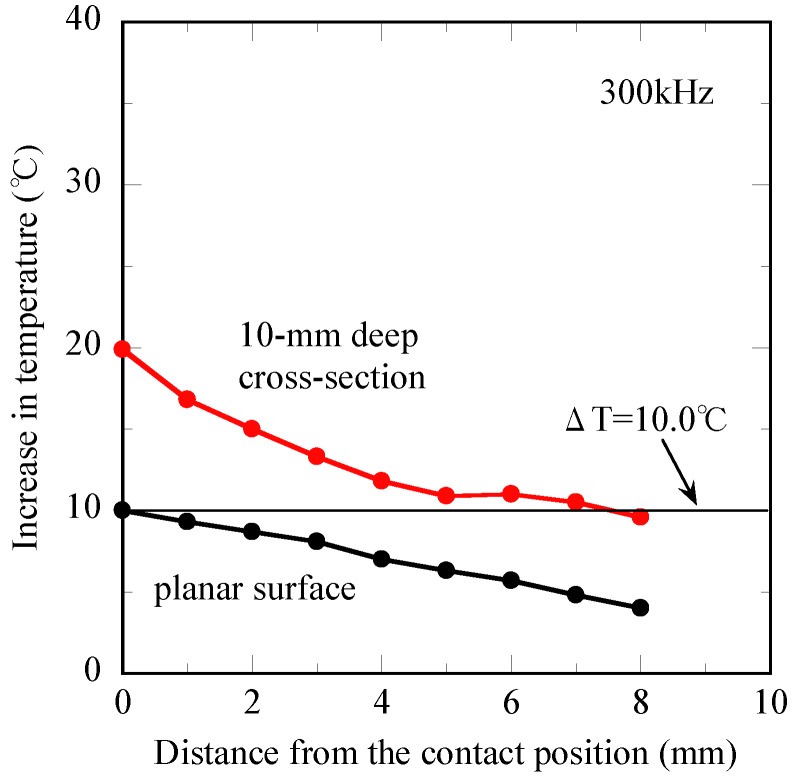
Changes in temperature of the tissue-mimicking phantom obtained at the planar surface and the 10-mm deep cross-section after 1,200 s *vs.* the distance from the contact position.

The relationship between the increase in temperature (ΔT) after 1,200 s and distance from the contact position is presented in Figure 7 for both the planar surface and 10-mm deep cross-section. In the 10-mm deep cross-section, the ΔT of the d = 0 mm position is 19.8 °C, and its value reached ΔT = 9.9 °C at the d = 8 mm position, showing the gradual decrease with the increasing distance from the contact position (d = 0 mm). However, the ΔT is 10.0 °C at the d = 0 mm position for the planar surface, while its value is at most 4.0 °C after the induction time of 1,200 s at the d = 8 mm position. Hence, the ΔT of the planar surface exhibits a lower value than those of the 10-mm deep cross-section. It is appropriate to consider that a significant heat dissipation takes place from the planar surface of the tissue-mimicking phantom to the ambient air.

### 3.3 Heat transfer Simulation Images of the Tissue-Mimicking Phantom

**Figure 8:**
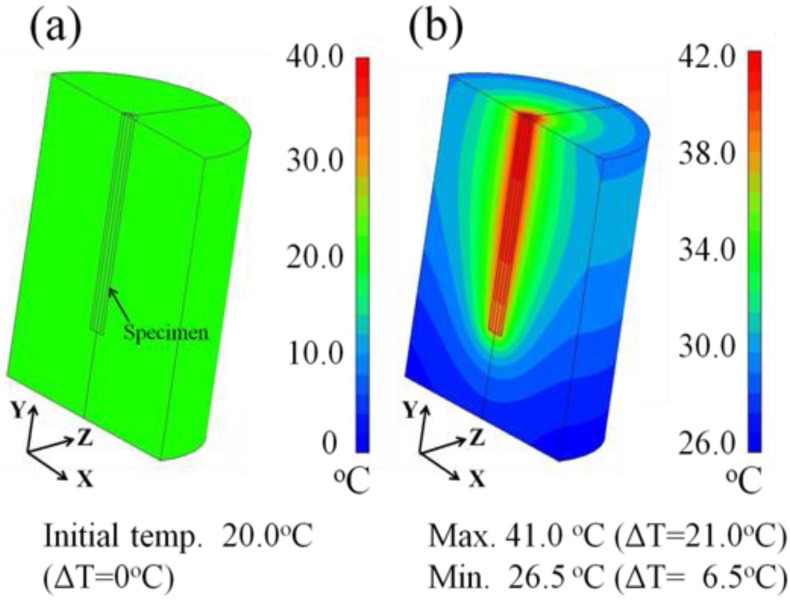
Heat transfer simulation images showing the temperature distribution at (**a**) 0 s and (**b**) after 1,200 s in the longitudinal section of the tissue-mimicking phantom.

Figure 8 shows the heat transfer simulation images of the tissue-mimicking phantom taken for the longitudinal section using a 180° simulation model. The temperature distribution is colored light green (Figure 8a) at the initial temperature of 20.0 °C, which corresponds to the ΔT = 0 °C for all planes. However, the generated heat transfers in the lateral direction (Figure 8b) during the induction time of 1200 s. Taking note of the longitudinal section, it spreads more widely around the middle position of the inserted 20-mm long specimen, while no significant heat transfer occurs to the underside of the specimen. It is concluded from these results that we cannot expect any ablation effect to the underside of the inserted ablation needle. 

**Figure 9:**
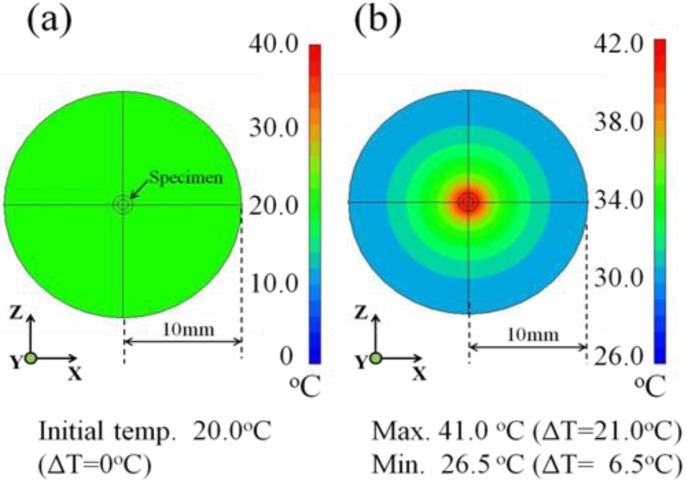
Heat transfer simulation images showing the temperature distribution at (**a**) 0 s and (**b**) after 1,200 s in the 10-mm deep cross-section of the tissue-mimicking phantom.

Figure 9 shows the heat transfer images of the tissue-mimicking phantom subjected to the θ = 0° specimen insertion for the 10-mm deep cross-section obtained after 1200 s. As seen in Figure 9a, the temperature distribution is colored light-green at the initial temperature of 20.0 °C. It is obvious from Figure 9b that the generated heat concentrically spreads during the induction time of 1200 s, showing the color transition as colored dark red, light green and light-gray blue and gray-blue. In addition, the simulated maximum temperature and the resultant ΔT value at the 10-mm deep cross-section are 41.0 °C and 21.0 °C, respectively, as denoted in the lower side of Figure 9b. Referring to the color display bar on the right side, the outer region of the concentric circle ranging from a 7-mm to 10-mm radius is colored gray-blue, suggesting that the temperature reaches 30.0 °C during the induction time of 1,200 s and the resultant ΔT value is approximately 10.0 °C. In comparison to Figure 7, we note that there is a good agreement between the experimental and simulated data for the 10-mm deep cross-section of the tissue-mimicking phantom. 

## 4. Conclusions

With the aim of estimating the applicability as a novel ablation therapy for oral cancer, the heat generation and transfer behaviors of a Ti-coated carbon steel rod were investigated using both water (15 mL) and a tissue-mimicking phantom in a high-frequency induction field at 300 kHz. The non-oriented heating property was achieved for the measurement using water (15 ml) by changing the inclination angles (θ = 0°, θ = 45° and θ = 90°) relative to the magnetic flux direction. In the heat transfer experiments employing the tissue-mimicking phantom, the ΔT value gradually decreased with the increasing distance from the contact position to an 8-mm distance for both the planar surface and 10-mm deep cross-section. However, the former exhibited significantly lower values than the latter through the distance up to 8-mm, probably due to the heat dissipation from the planar surface to the ambient air. The concentric heat spread in the lateral direction was visualized by the heat transfer simulation images. Furthermore, no significant heat transfer was found to occur to the underside of the specimen, which is situated in the longitudinal direction. Based on these results, it seems likely that the Ti-coated carbon steel rod possesses a high potential as an ablation needle for treating superficial oral cancer. 
